# Development and validation of a preoperative “difficulty score” for laparoscopic transabdominal adrenalectomy: a multicenter retrospective study

**DOI:** 10.1007/s00464-021-08678-6

**Published:** 2021-08-17

**Authors:** Laura Alberici, Alessandro M. Paganini, Claudio Ricci, Andrea Balla, Zeno Ballarini, Monica Ortenzi, Giovanni Casole, Silvia Quaresima, Guido Di Dalmazi, Pietro Ursi, Marie Sophie Alfano, Saverio Selva, Riccardo Casadei, Carlo Ingaldi, Giovanni Lezoche, Mario Guerrieri, Francesco Minni, Guido Alberto Massimo Tiberio

**Affiliations:** 1 Department of Internal Medicine and Surgery (DIMEC), Alma Mater Studiorum, University of Bologna, Bologna, Italy; 2 Division of Pancreatic Surgery, IRCCS, Azienda Ospedaliero Universitaria di Bologna, Bologna, Italy; 3 Bariatric Surgery Unit, Department of General Surgery and Surgical Specialties “Paride Stefanini”, AOU Policlinico Umberto I, Sapienza University of Rome, Rome, Italy; 4 Surgical Clinic, Department of Clinical and Experimental Sciences, The University of Brescia at ASST Spedali Civili di Brescia, Brescia, Italy; 5 Clinica Chirurgica Generale e d’Urgenza, AOU Umberto I-Lancisi-Salesi, Ancona, Italy; 6 Unit of Endocrinology and Diabetes Prevention and Care, S.Orsola-Malpighi Hospital, Bologna, Italy; 7 Policlinico S.Orsola-Malpighi, Alma Mater Studiorum-Università di Bologna, Via Massarenti n.9, 40138 Bologna, Italy

**Keywords:** Laparoscopic adrenalectomy, Difficulty score, Postoperative complications

## Abstract

**Background:**

A difficulty score for laparoscopic adrenalectomy (LA) is lacking in the literature. A retrospective cohort study was designed to develop a preoperative “difficulty score” for LA.

**Methods:**

A multicenter study was conducted involving four Italian tertiary centers for adrenal disease. The population was randomly divided into two subsets: training group and validation one. A multicenter study was undertaken, including 964 patients. Patient, adrenal lesion, surgeon’s characteristics, and the type of procedure were studied as potential predictors of target events. The operative time (pOT), conversion rate (cLA), or both were used as indicators of the difficulty in three multivariate models. All models were developed in a training cohort (70% of the sample) and validated using 30% of patients. For all models, the ability to predict complicated postoperative course was reported describing the area under the curve (AUCs). Logistic regression, reporting odds ratio (OR) with *p*-value, was used.

**Results:**

In model A, gender (OR 2.04, *p* = 0.001), BMI (OR 1.07, *p* = 0.002), previous surgery (OR 1.29, *p* = 0.048), site (OR 21.8, *p* < 0.001) and size of the lesion (OR 1.16, *p* = 0.002), cumulative sum of procedures (OR 0.99, *p* < 0.001), extended (OR 26.72, *p* < 0.001) or associated procedures (OR 4.32, *p* = 0.015) increased the pOT. In model B, ASA (OR 2.86, *p* = 0.001), lesion size (OR 1.20, *p* = 0.005), and extended resection (OR 8.85, *p* = 0.007) increased the cLA risk. Model C had similar results to model A. All scores obtained predicted the target events in validation cohort (OR 1.99, *p* < 0.001; OR 1.37, *p* = 0.007; OR 1.70, *p* < 0.001, score A, B, and C, respectively). The AUCs in predicting complications were 0.740, 0.686, and 0.763 for model A, B, and C, respectively.

**Conclusion:**

A difficulty score based on both pOT and cLA (Model C) was developed using 70% of the sample. The score was validated using a second cohort. Finally, the score was tested, and its results are able to predict a complicated postoperative course.

**Supplementary Information:**

The online version contains supplementary material available at 10.1007/s00464-021-08678-6.

Since it was first described in 1992 by Gagner et al. [1–4], laparoscopic adrenalectomy (LA) has become the standard treatment for most adrenal lesions. Nevertheless, it requires appropriate knowledge of abdominal and retroperitoneal anatomy, proven expertise, and technical skills to avoid surgical complications of the surgical procedure [5–7]. According to Guidelines [8, 9], LA’s indications include small to medium-sized (≤ 6 cm) benign adrenal tumors, both functioning and non-functioning, whereas a laparoscopic approach to suspected or proven adrenal cortical carcinoma and large adrenal masses is yet controversial. At present, several studies have compared the outcomes of different approaches to adrenalectomy [10–16] without remarkable differences. On the other hand, some differences between the first and second generation of laparoscopic surgeons have been demonstrated, remarking a tutor’s role during the training period [17]. However, a “difficulty score” for LA is lacking in the literature. The aim of this study was to develop a preoperative “difficulty score,” analyzing a large series of patients who underwent LA in high-volume tertiary centers.

## Methods

A multicenter retrospective observational study was undertaken at the Departments of General Surgery of Bologna (Alma Mater Studiorum—Policlinico S. Orsola-Malpighi), Brescia (Università di Brescia—ASST Spedali Civili), Ancona (Università Politecnica delle Marche), and Roma (Università La Sapienza). All of them are referral centers for adrenal surgery in Italy and have prospectively maintained databases. For each case, the indication for surgical treatment was approved by a multi-disciplinary team, including surgeons, endocrinologists, radiologists, and pathologists dedicated to adrenal diseases. The anesthesiologist evaluation completed the preoperative risk stratification. Data were extrapolated from prospectively collected databases and managed according to Institutional rules, with the patient's consent. All patients undergoing LA from January 1994 to September 2020 were included in the study. Patients who underwent adrenalectomy with an open approach, exploratory laparoscopy, or surgery for recurrence of disease after LA were a priori excluded. The authors screened 976 records, and 12 patients were excluded for incomplete data. In the final analysis, the authors included 964 patients. For each record, the following perioperative data were extracted: characteristics of the patient (gender, age, body mass index, ASA score, comorbidities, previous abdominal surgery, presence of symptoms); characteristics of the adrenal lesion (side, size, presumptive diagnosis of functioning or non-functioning and benign or malignant lesion based on clinical–radiological data); characteristics of the surgeon (cumulative sum of procedures performed, distinction in a junior or senior surgeon [17]); characteristics of the planned procedure (need for extended resection or other surgical procedures); intraoperative data (operative time, laparoscopic approach with or without need for conversion, blood loss); postoperative data such as complications according to Clavien–Dindo classification [18], (CDC) resumption of enteral feeding, need for intensive care in ICU, length of ICU stay, length of hospitalization, 90 days mortality, and histological diagnosis. It should be noted that the cumulative sum of procedures (CUSUM) was described for each surgeon as a progressive ordinal number. Thus, CUSUM reflects the experience of each operator at the time of each procedure.

### Statistical analysis

All categorical variables were reported as frequencies and percentages, whereas continuous variables were reported as the median and interquartile range (IQR). An operative time above the 75th percentile (pOT) or conversion to open surgery (cLA) was considered indicative of difficulty. A complicated postoperative course > II CDC class was used to test the utility of difficulty scores. Three predictive models were built: (1) model A, in which all preoperative factors predicting pOT were studied; (2) model B, in which all preoperative factors predicting cLA were studied; (3) model C, in which all preoperative factors predicting both the events were studied. The analysis was carried out in three steps. Firstly, preoperative variables were pre-selected using the least absolute shrinkage and selection operator (LASSO) method [19]. For the subsequent two steps, the cohort was divided into a training (including 70% of patients) and a validation cohort (including the remaining 30%). Patients were casually distributed, independently from the center and the date of surgery, in the two subsets by a random number generator to avoid any time-depending bias. Secondly, all models were analyzed in a training cohort (70% of patients). All models were graphically represented by a nomogram [20] and were converted into a score. A validation was obtained using the remaining 30% of patients (validation cohort) in the third step. Calibration was made using the post-regression estimation of the marginal values.

For each score, the diagnostic accuracy (AUC) was described and interpreted as follows: excellent > 0.9, good between 0.8–0.9, fair between 0.7 and 0.8, poor between 0.6 and 0.7, and failed < 0.6. The three models' utility was tested to predict a severe complicated postoperative. All analysis was made using logistic regression reporting odds ratio (OR) and standard error (SE). STATA 14 software (StataCorp.) 2011 was used to carry out all analyses. All details were exhaustively reported in the Supplementary methods.

## Results

The entire cohort included 964 patients undergoing laparoscopic adrenalectomy with a transperitoneal approach. The breakdown by centers was the following: 51.7% of patients (498) from Ancona, 26.1% (252) from Bologna, 12.9% (124) from Brescia, and 9.3% (90) from Rome. One senior surgeon (AMP) participated in the University of Ancona case series from 1994 to 2002 and in the University of Rome case series from 2002 to 2020. Preoperative data are described in Supplementary Table 1.

The cohort included 577 (59.9%) female and 387 (40.1%) male patients with a median age of 55 years (43–64, IQR) and a BMI of 26 kg/m^2^ (23–29, IQR). The distribution of the ASA score was as follows: I for 109 patients (11.3%), II for 526 patients (54.6%), III for 318 patients (33.0%), IV for 11 patients (1.1%). Almost half of the patients (41.1%) have undergone previous abdominal surgery, and 52.8% were symptomatic at diagnosis.

The suspected preoperative diagnosis was a benign non-functioning cortical lesion in 291 cases (30.2%); a benign functioning cortical lesion in 397 cases (41.2%), 200 of which (20.8%) associated with Conn disease, 137 (14.2%) associated with Cushing disease or syndrome, 57 (5.9%) associated with “mild autonomous cortisol excess” syndrome, 5 (0.5%) associated with mixed secretion of mineralocorticoids and glucocorticoids, and 3 (0.3%) associated with secretion of androgens. In 190 cases (19.7%), the preoperative diagnosis was pheochromocytoma and in 86 cases (8.9%), malignant lesion.

Overall, the series included 458 right (47.5%), 479 left (49.7%), and 27 bilateral (2.8%) adrenalectomies. The median overall tumor size was 3.5 cm (2.5–5, IQR). The median cumulative sum of procedures per surgeon was 146 (52–269, IQR). In 144 cases (14.9%), surgery was carried out by a junior surgeon and in 820 cases (85.1%) by a senior surgeon. Only in 10 (1.1%) and 25 (2.6%) cases, extended resection or associated surgical procedures were planned, respectively.

The postoperative results are summarized in Supplementary Table 2.

The median operative time was 100 (75–140) minutes, and the conversion rate was 5.2% (50 cases). In the majority of patients (945, 98.1%), postoperative complications were absent or mild (grade I or II, according to Clavien–Dindo classification). Twelve patients (1.2%) required percutaneous radiological procedures, and 5 (0.5%) required readmission to the ICU as a consequence of an adverse event (grade III and IV according to Clavien–Dindo, respectively). The reoperation rate was 0.6% (6), and the 90-day mortality rate was 0.2% (2 cases). Perioperative blood transfusions were required in 3.5% of patients. The median duration of hospital stay was 4 days (3–6, IQR). Histology of the removed lesions showed that 56.6% were benign cortical lesions, 19.7% were medullary lesions, 6.5% were adrenal metastasis, 5.2% were myelolipomas, 4.4% were adrenocortical carcinomas, 2.9% were cystic lesions, 2.9% were collision tumors, 1.2% other malignancy, and 0.5% were inflammatory lesions.

### First step: preselection of the covariates

The covariates potentially predicting model A (pOT) were age, gender, presence of symptoms, clinical–radiological diagnosis, side of the lesion, BMI, ASA score, previous surgery, lesion size, type of surgeon, CUSUM, and associated or extended surgery. The optimal Lambda value was 0.037. The selection process is shown in Supplementary Fig. 1, panel A. The covariates potentially predicting model B (cLA) were the presence of symptoms, clinical–radiological diagnosis, side of the lesion, BMI, ASA score, previous surgery, lesion size, type of surgeon, and associated or extended surgery. The optimal Lambda value was 6.113. The selection process is shown in Supplementary Fig. 1, panel B.

The covariates potentially predicting model C (pOT or cLA) were the same of model A. The optimal Lambda value was 0.034. The selection process is shown in Supplementary Fig. 1, panel C.

### Second step: analysis on training cohort

The multivariate analysis on a cohort of 679 patients (70% of the total) is reported in Tables 1, 2, and 3 for models A, B, and C, respectively.

**Table 1:** Factors predicting an operative time over 75th percentile (training set, *N* = 679)

Parameters	OR ± SE	*p*-value	Step exclusion
Gender (Female vs. male)	2.04 ± 0.45	0.001	Final
Age (for each year)	0.98 ± 0.01	0.189	4th
BMI (for each Kg/m^2^)	1.07 ± 0.02	0.002	Final
ASA score (for each class)	1.29 ± 0.22	0.126	5th
Previous surgery (No vs. yes)	1.53 ± 0.33	0.048	Final
Symptoms (No vs. yes)	0.72 ± 0.16	0.146	3rd
Clinical and radiological diagnosis			2nd
Non-functioning benign lesion	Referent		
Functioning benign lesion	1.21 ± 0.38	0.516	
Pheochromocytoma	1.71 ± 0.63	0.142	
Malignant lesion	1.19 ± 0.55	0.690	
Side			Final
Right	Referent		
Left	1.12 ± 0.23	0.590	
Bilateral	21.8 ± 17.6	< 0.001	
Size (for each cm)	1.16 ± 0.06	0.002	Final
Type of surgeon (Junior vs. senior)	0.97 ± 0.04	0.468	1st
Cumulative sum of procedures	0.99 ± 0.01	< 0.001	Final
Extended resection planned (No vs. yes)	26.72 ± 24.67	< 0.001	Final
Others surgical procedures planned (No vs. yes)	4.32 ± 2.61	0.015	Final

*OR* odds ratio, *SE* standard error, *BMI* Body Mass Index, *ASA* American Society of Anesthesiologists score

**Table 2:** Factors predicting conversion to open surgery (training set, *N* = 679)

Parameters	OR ± SE	*p*-value	Step exclusion
BMI (for each Kg/m^2^)	1.01 ± 0.04	0.900	2nd
ASA score (for each class)	2.86 ± 0.89	0.001	Final
Previous surgery (No vs. yes)	1.41 ± 0.55	0.376	5th
Symptoms (No vs. yes)	1.03 ± 0.55	0.994	1st
Diagnosis			7th
Non-functioning benign lesion	Referent		
Functioning benign lesion	0.41 ± 0.21	0.082	
Pheochromocytoma	0.23 ± 0.18	0.063	
Malignant lesion	2.52 ± 1.27	0.068	
Side			4th
Right	Referent		
Left	0.71 ± 0.29	0.415	
Bilateral	2.06 ± 1.93	0.440	
Size (for each cm)	1.20 ± 0.07	0.005	Final
Type of surgeon (Junior vs. senior)	1.05 ± 0.07	0.479	3rd
Extended resection planned (No vs. yes)	8.85 ± 7.20	0.007	Final
Others surgical procedures planned (No vs. yes)	2.64 ± 2.18	0.239	6th

*OR* odds ratio, *SE* standard error, *BMI* Body Mass Index, *ASA* American Society of Anesthesiologists score

**Table 3:** Factors predicting operative time over 75th percentile or conversion to open surgery (training set, *N* = 679)

Parameters	OR ± SE	*p*-value	Step exclusion
Gender (Female vs. male)	1.87 ± 0.39	0.003	Final
Age (for each year)	0.98 ± 0.01	0.117	3rd
BMI (for each Kg/m^2^)	1.07 ± 0.02	< 0.001	Final
ASA score (for each class)	1.33 ± 0.21	0.074	5th
Previous surgery	1.56 ± 0.32	0.032	Final
Symptoms (No vs. yes)	0.72 ± 0.15	0.113	4th
Diagnosis			2nd
Non-functioning benign lesion	Referent		
Functioning benign lesion	1.16 ± 0.36	0.619	
Pheochromocytoma	1.55 ± 0.54	0.217	
Malignant lesion	1.43 ± 0.60	0.400	
Side			Final
Right	Referent		
Left	1.01 ± 0.21	0.929	
Bilateral	15.15 ± 11.26	< 0.001	
Size (for each cm)	1.15 ± 0.05	0.002	Final
Type of surgeon (Junior vs. Senior)	0.98 ± 0.03	0.580	1st
Cumulative sum of procedures	0.99 ± 0.01	< 0.001	Final
Extended resection planned (No vs. yes)	12.24 ± 10.50	0.003	Final
Others surgical procedures planned (No vs. yes)	4.67 ± 2.75	0.009	Final

*OR* odds ratio, *SE* standard error, *BMI* Body Mass Index, *ASA* American Society of Anesthesiologists score

**Fig. 1:**
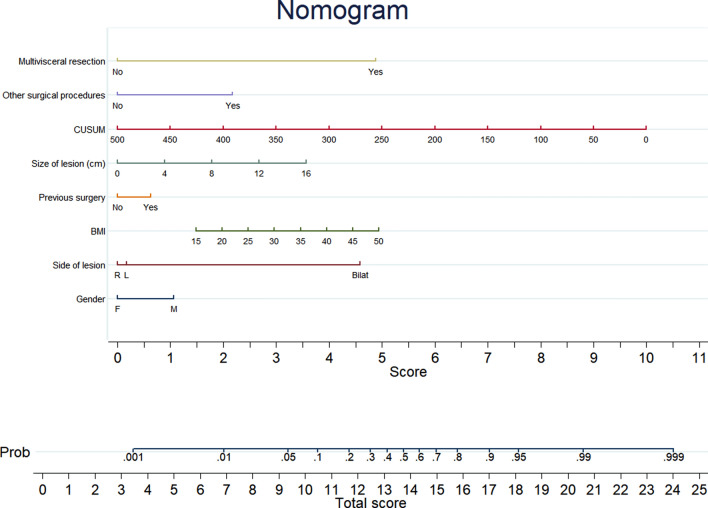
Nomogram of model A (operative time over 75th percentile)

**Fig. 2:**
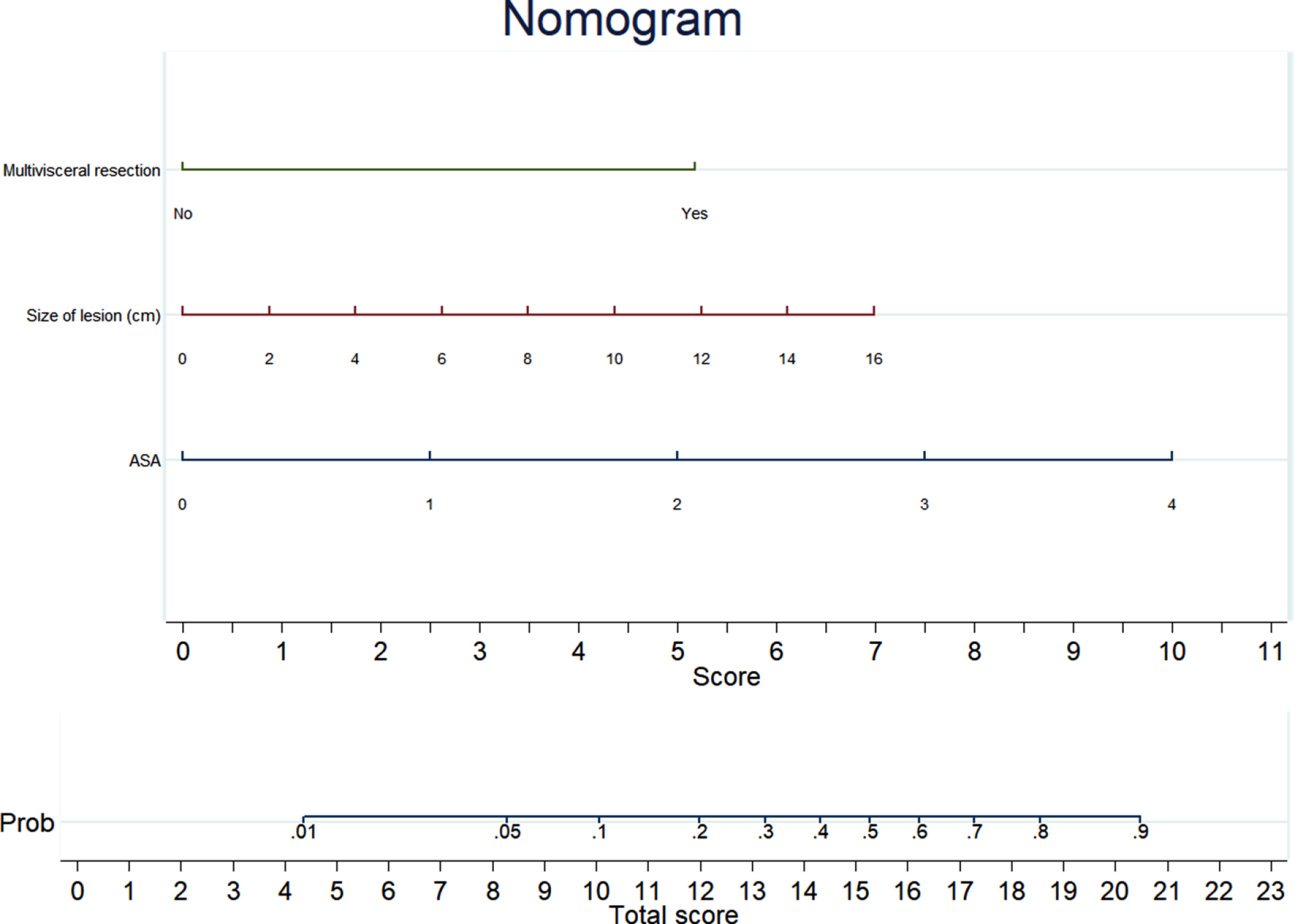
Nomogram of model B (converted procedure)

**Fig. 3:**
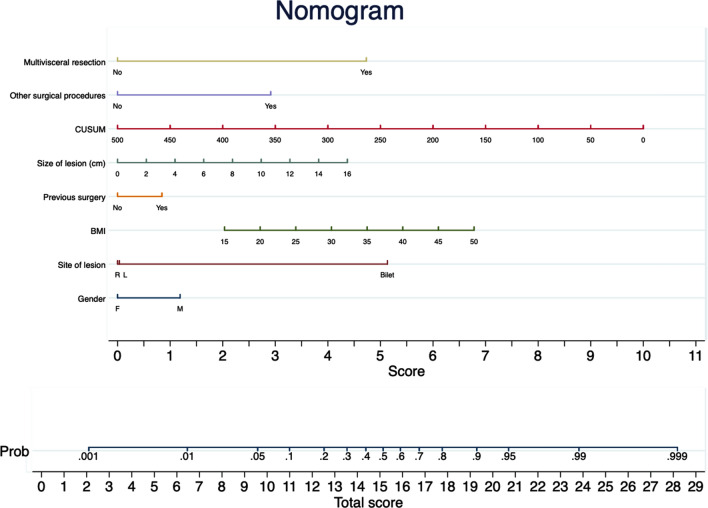
Nomogram of model C (operative time over 75th percentile or converted procedure)

In model A (pOT), male gender (OR 2.04, *p* = 0.001), BMI (OR 1.07 for each Kg/m^2^, *p* = 0.002), previous surgery (OR 1.29, *p* = 0.048), bilateral site of lesions (OR 21.8, *p* < 0.001), size of the lesion (OR 1.16 for each cm, *p* = 0.002), cumulative sum of procedures (OR 0.99, *p* < 0.001), extended resection (OR 26.72, *p* < 0.001), and associated surgical procedures (OR 4.32, *p* = 0.015) significantly influenced the operative time. The nomogram obtained from model A is shown in Fig. 1. Score A ranges from a minimum of 0 to a maximum of 33.5 points. In model B (cLA), ASA score (OR 2.86, *p* = 0.001), size of the lesion (OR 1.20 for each cm, *p* = 0.005), and the need for extended resection (OR 8.85, *p* = 0.007) significantly increased the risk of conversion. The nomogram obtained from model B is shown in Fig. 2. Score B ranges from a minimum of 0 to a maximum of 23 points. In model C (pOT or cLA), male gender (OR 1.87, *p* = 0.003), BMI (OR 1.07 for each Kg/m^2^, *p* = 0.001), previous surgery (OR 1.56, *p* = 0.032), bilateral site of lesions (OR 15.15, *p* < 0.001), size of lesion (OR 1.15 for each cm, *p* = 0.002), cumulative sum of procedures (OR 0.99, *p* < 0.001), extended resection (OR 12.24, *p* = 0.003), and associated surgical procedures (OR 4.67, *p* = 0.009) were found to have significant relationship with the operative time or the conversion rate. The nomogram obtained from model C is shown in Fig. 3. Score C ranges from a minimum of 0 to a maximum of 33.5 points. The AUCs were 0.833 ± 0.016, 0.710 ± 0.048, and 0.809 ± 0.019 fo Score A, B, and C, respectively (Supplementary Fig. 2 panel A, B, and C).

### Third step: analysis of the validation and test cohort

Scores A, B, and C were validated on a cohort of 285 patients (30% of the total).

Score A has been proven to significantly predict an operative time extension's increased risk beyond 140 min (OR 1.99 ± 0.19 for each point, *p* < 0.001). Supplementary Fig. 3 panel A shows the curve’s trend representing Score A. Score B has been proven to significantly predict the increased risk of conversion (OR 1.37 ± 0.16 for each point, *p* = 0.007). Supplementary Fig. 3 panel B shows the curve trend representing Score B. Score C has been proven to significantly predict the increased risk of an operative time extension beyond 140 min or conversion (OR 1.70 ± 0.13 for each point, *p* < 0.001). Supplementary Fig. 3 panel C shows the trend of the curve representing Score C. The AUCs of the three models were 0.820 ± 0.015 for score C, 0.819 ± 0.015 for score A, and 0.6333 ± 0.0208 for score B (Supplementary Fig. 4 panel A, B, and C).

**Fig. 4:**
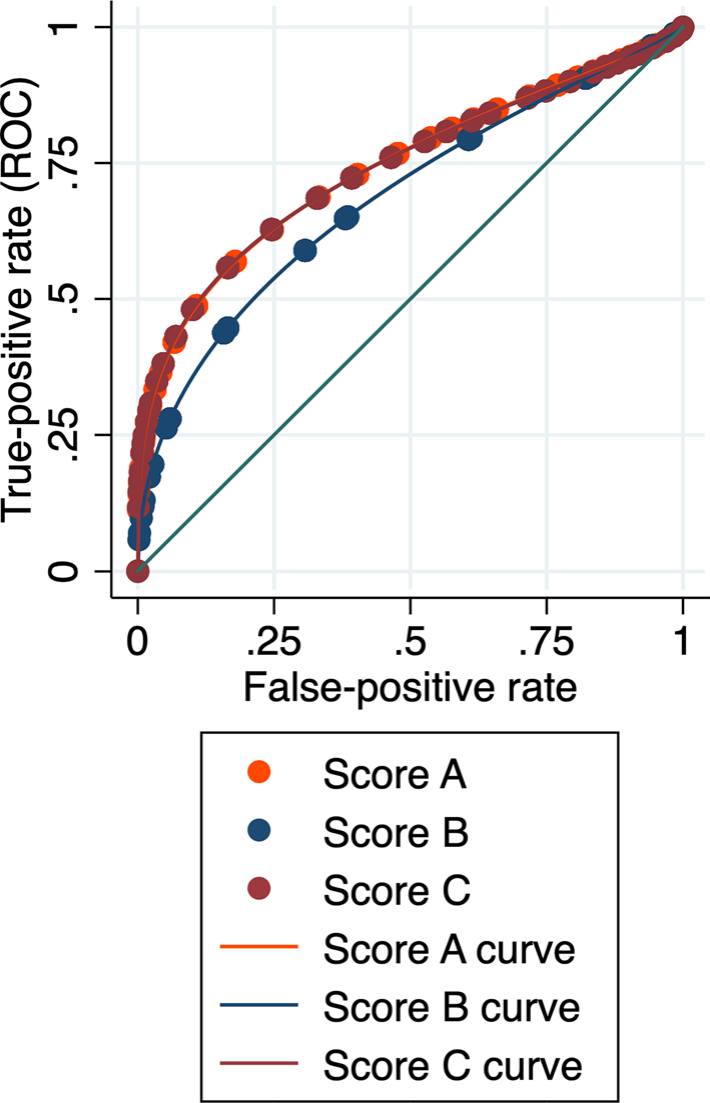
Receiver operating characteristic (ROC) curves for model A, B, and C in predicting complicated postoperative course (Clavien–Dindo class > 2)

All three models (A, B, and C) were significantly related (*p* < 0.001) to a complicated postoperative course defined as CDC class > II: OR 1.29 ± 0.81, 1.72 ± 0.23, and 1.25 ± 0.06 for Score A, B, and C, respectively. The AUCs values in predicting severe postoperative complications were 0.740 ± 0.071, 0.686 ± 0.069, and 0.763 ± 0.415 for Model A, B, and C, respectively (Fig. 4).

## Discussion

The present study demonstrated that some preoperative parameters are useful to predict the difficulty of LA. In this study, 964 laparoscopic adrenalectomies performed in four high-volume centers are described. To our knowledge, this is one of the largest cohorts in which the difficulty of LA was evaluated. Similar to other experiences [21–23], the difficulty was measured using the operative time and conversion rate. The severe postoperative complication rate was used to confirm the utility of the scores. The perioperative transfusion rate was not used as an indicator of difficulty because transfusions were relatively rare events (3.5%) related to the conversion rate. Thus, a model based on transfusion rate could overlap the model based on conversion rate without a gain in statistical power. Three separate models were developed: one for the operative time (A), one for the conversion (B), and one for both (C). Each model was studied in a training cohort (70% of the sample) and confirmed in a validation cohort (30% of the sample). The results observed in the training cohort were those expected based on literature data both for operative time [14–17, 23–28] and conversion rate [20–22]. Indeed, male gender, high BMI, previous abdominal surgery, bilaterality of lesion, size of the lesion [29–32], associated surgical procedures, and need for extended resections prolonged the operative time, increasing the probability of overcoming the 75th percentile. The surgeon’s experience, on the contrary, reduced the probability of a prolonged operative time. The lesion’s size, the ASA score, and the need for extended resection increased conversion probability. All three models are clinically plausible, easily computable using a simple nomogram (Figs. 1, 2, 3), and provide a numerical score related to the target events' probability.

Nevertheless, models A and C have good accuracy, whereas B has fair accuracy. The models were validated and calibrated using the second cohort of patients, confirming the results’ statistical plausibility. In the validation cohort, the AUCs of models A and C were confirmed to be good, correctly classifying eight patients every ten tested. On the contrary, model B was not so accurate, correctly classifying only six patients every ten. Therefore, the most useful model to predict a difficult LA seems to be model C because it demonstrated a high AUC and, at the same time, the ability to predict both adverse events (conversion or prolonged operative time). This finding did not surprise: a model based only on the conversion rate could not include all “difficult” procedures. In other words, not all challenging procedures were converted even if performed by a skilled laparoscopic surgeon. A second interesting result was that both A and C models take into consideration many types of factors. Indeed, the scores included both patient and disease characteristics, not forgetting the type of procedures planned and the surgeon’s experience. The score could practically help the chief surgeon plan the procedures and proper patient counseling. The models’ utility was further underlined by the correlation between A and C scores with the probability of a complicated postoperative course.

This study has some limitations. First, the design of the study was retrospective. However, all databases come from high-volume centers and are prospectively maintained. Moreover, all postoperative data suggested the high quality of surgery with very low conversion and postoperative complication rates. A second limitation was the large enrollment period and the relative changes in surgeon training and medical devices over the last 20 years. According to the period in which they were trained, the bias was partially mitigated and studied by dividing the surgeons into first- and second-generation surgeons [17].

Moreover, all time-depending bias, such as different distribution of significant factors, was overcome by the study design. Indeed, the entire cohort was randomly divided, independently from the center and date of surgery, in a training and validation cohort. A third limitation was the low number of target events (converted or prolonged procedures) used to build the scores. Indeed, the low number of events could affect the robustness of multivariate models.

Nevertheless, the LASSO approach's use solved the overfitting, reducing, when necessary, the number of covariates for the multivariates analysis. A further limitation was the applicability of the difficulty score only in transperitoneal approaches. All involved centers performed LA using the anterior or lateral transperitoneal approach, and for this reason, the model was not validated for the retroperitoneal approach.

In conclusion, we reported, validated, and tested a difficulty score for laparoscopic adrenalectomy for the first time. The obtained models, particularly model C, could predict two critical events: conversion to open surgery and prolonged operative time. The score for each model corresponds to the probability that the target event may occur. It is simple to calculate preoperatively, practical to use, and could be used not only for the surgical team’s choice but also to predict and avoid a complicated postoperative course. An external validation would be recommended to confirm these results further.

## Supplementary Information

Below is the link to the electronic supplementary material.

Supplementary file1 (DOCX 15 kb)

Supplementary file2 (DOCX 56807 kb)
