# Supplementary material for: Development and validation of a preoperative “difficulty score” for laparoscopic transabdominal adrenalectomy: a multicenter retrospective study
Source: Surg Endosc. 2021 Aug 17;36(5):3549–57. doi: 10.1007/s00464-021-08678-6 (PMC9001553; doi:10.1007/s00464-021-08678-6)
Supplement: Supplementary file 1 — Supplementary file1 (DOCX 15 kb) [file 464_2021_8678_MOESM1_ESM.docx]

**Supplementary Methods**

*Terminology and definitions*

We considered symptomatic all patients with clinical signs or symptoms related to adrenal hormonal secretion or mass effect at the time of diagnosis. We defined *Senior surgeons* the "first generation" of laparoscopic surgeons, which performed their initial procedures at the time of the advent of laparoscopy without receiving specific training or supervision. We identified as *Junior surgeons* the "second generation" of laparoscopic surgeons, which carried out their intervention after their fellowship training in the era of laparoscopic surgery and under their mentor's supervision.^1^

The cumulative sum of procedures was defined as the cumulative sum of consecutive procedures performed by each surgeon. Operative time was measured as the time, in minutes, between the surgical incision and the skin wound suture. Anesthesiological induction time and patient's placement on the operative table were not included in the operative time. Extended resection was defined as en bloc resection for organs or structures macroscopically adherent to the primary tumor according to oncological principles such as sufficient resection margin and intact tumor capsule (e.g., nephrectomy or hepatic resection).

On the contrary, associated procedures were defined as other surgical treatments of conditions not associated with adrenal disease (e.g., correction of umbilical hernia or cholecystectomy for lithiasis).

*Statistical analysis*

All categorical variables were reported as frequencies and percentages, whereas continuous variables were reported as median and interquartile range (IQR). All preoperative variables related to patient, disease, planned type of surgery, and surgeons were used as potential predictors of difficulty. An operative time above the 75^th^ percentile (pOT) or conversion to open surgery (cLA) were considered as indicative of difficulty. Complicated postoperative course > II CDC class was used to test the utility of difficulty scores. All other postoperative results were reported for descriptive purposes but not analyzed.

We built three predictive models: 1) model A, in which all preoperative factors predicting pOT were studied; 2) model B, in which all preoperative factors predicting cLA were studied; 3) model C, in which all preoperative factors predicting both the events were studied. The analysis was carried out for each model (A, B, and C) in three consecutive steps. In the first step, a preselection of preoperative variables was made on the entire cohort of patients. Considering the presence of a large number of regressors and the high risk of over-fitting due to the small number of events, we preselected the variables to include in the models using the least absolute shrinkage and selection operator (LASSO) method. ^2^

For the subsequent two steps, the cohort was divided into two subsets: a training cohort (including 70% of patients) and a validation cohort (including the remaining 30%). Patients were casually distributed in the two subsets by a random number generator. In the second step, for all three predictive models (Model A, B, and C), a multivariate analysis was carried out on the training cohort using only the preselected covariates. This analysis was conducted using logistic regression with backward elimination method. The logistic regression results were expressed as odds ratio (OR) and standard error (SE). A p-value of less than 0.05 (two-sided) was considered statistically significant. All variables independently significant at the multivariate analysis were selected to create a nomogram. The nomogram was generated according to the mathematical algorithm proposed by Kattan et al.^3^ Through this algorithm, the beta logistic regression coefficients of the selected variables can be converted into a numerical score from 1 to 10. The cumulative score for all the variables corresponds to the probability of the "target event." On this assumption, three nomograms (nomogram A, B, and C) and three related scores (Score A, B, and C) were created, in order to predict the risk of an pOT (model A), of cLA (model B) or both the events (model C) respectively. For each score, the diagnostic accuracy (AUC) was described. The AUC was considered excellent for values between 0.9-1, good for values between 0.8-0.9, fair for values between 0.7-0.8, poor for AUC values between 0.6-0.7, and failed for values between 0.5-0.6.

In the third step, we performed the scores' validation and calibration by studying the second subset of patients (validation and test cohort). The validation was performed by regressing each score on the respective target events by using logistic regression. The calibration was made using the post-regression estimation of the marginal values​. All possible score values ​​were tested. Once again, for each score, the diagnostic accuracy (AUC) was described.

Finally, the utility of the three models were tested to predict a severe complicated postoperative course by using logistic regression in entire cohort. STATA 14 software (StataCorp.) 2011 was used to carry out all analyses.

References

1. Alberici L, Ricci C, Ingaldi C, et al. The learning curve for the second generation of laparoscopic surgeons: lesson learned from a large series of laparoscopic adrenalectomies. Surg Endosc. 2020 Epub head of print.
2. Tibshirani R. Regression shrinkage and selection via the Lasso. J R Stat Soc Series B Stat Methodol 1996; 58:267–288.
3. Kattan MW, Eastham JA, Stapleton AM, et al. A preoperative nomogram for disease recurrence following radical prostatectomy for prostate cancer. J Natl Cancer Inst. 1998; 90:766-771.
