# Supplementary material for: Development and validation of a preoperative “difficulty score” for laparoscopic transabdominal adrenalectomy: a multicenter retrospective study
Source: Surg Endosc. 2021 Aug 17;36(5):3549–57. doi: 10.1007/s00464-021-08678-6 (PMC9001553; doi:10.1007/s00464-021-08678-6)
Supplement: Supplementary file 2 — Supplementary file2 (DOCX 56807 kb) [file 464_2021_8678_MOESM2_ESM.docx]

| **Pre-operative parameters** | **N or median (% or IQR)** |
| --- | --- |
| **Gender**  Female  Male | 577 (59.9)  387 (40.1) |
| **Age** (years) | 55 (43 – 64) |
| **BMI** (Kg/m^2^) | 26 (23 – 29) |
| **ASA score**  I  II  III  IV | 109 (11.3)  526 (54.6)  318 (33) 11 (1.1) |
| **Previous surgery**  No  Yes | 568 (58.9)  396 (41.1) |
| **Symptoms**  No  Yes | 455 (47.2)  509 (52.8) |
| **Clinical and radiological diagnosis**  Non-functioning benign lesions  Functioning benign lesions  Phaeochromocytomas  Malignant lesions | 291 (30.2)  397 (41.2)  190 (19.7)  86 (8.9) |
| **Side**  Right  Left  Bilateral | 458 (47.5)  479 (49.7)  27 (2.8) |
| **Size of lesion** (cm) | 3.5 (2.5 – 5) |
| **Cumulative number of procedures** | 146 (52 – 269) |
| **Type of Surgeon**  Junior  Senior | 144 (14.9)  820 (85.1) |
| **Extended resection planned**  No  Yes | 954 (98.9)  10 (1.1) |
| **Others surgical procedures planned**  No  Yes | 939 (97.4)  25 (2.6) |

**Supplementary Table 1- Characteristics of included 964 patients**

**Legend:** IQR= interquartile range; BMI= Body Mass Index; ASA= American Society of Anesthesiologists score.

**Supplementary Table 2- Postoperative results**

| **Post-operative results** | **N or median (% or IQR)** |
| --- | --- |
| **Operative time** (minutes) | 100 (75 – 140) |
| **Conversion**  No  Yes | 914 (94.8)  50 (5.2) |
| **Complications (CDC)**^18^  No  I  II  III  IV  V | 388 (40.3)  514 (53.3)  43 (4.5)  12 (1.2)  5 (0.5)  2 (0.2) |
| **Reoperations**  No  Yes | 958 (99.4)  6 (0.6) |
| **Peri-operative transfusions**  No  Yes | 930 (96.5)  34 (3.5) |
| **Hospital stay** (days) | 4 (3-6) |
| **Histological diagnosis**  Cortical benign lesion  Medullary lesion  Myelolipoma  Cortical malignant lesion  Secondary malignant lesions  Benign cystic lesions  Other malignant lesions  Inflammatory lesion  Collision tumor | 546 (56.6)  190 (19.7)  50 (5.2)  42 (4.4)  63 (6.5)  28 (2.9)  12 (1.2)  5 (0.5)  28 (2.9) |

**Legend**: CDC= Clavien Dindo classification system^18^.

**Supplementary Figures 1 -** Least absolute shrinkage and selection operator (LASSO) method to preselect the variables- **Panel A-** Model A, operative time over 75^th^ percentile


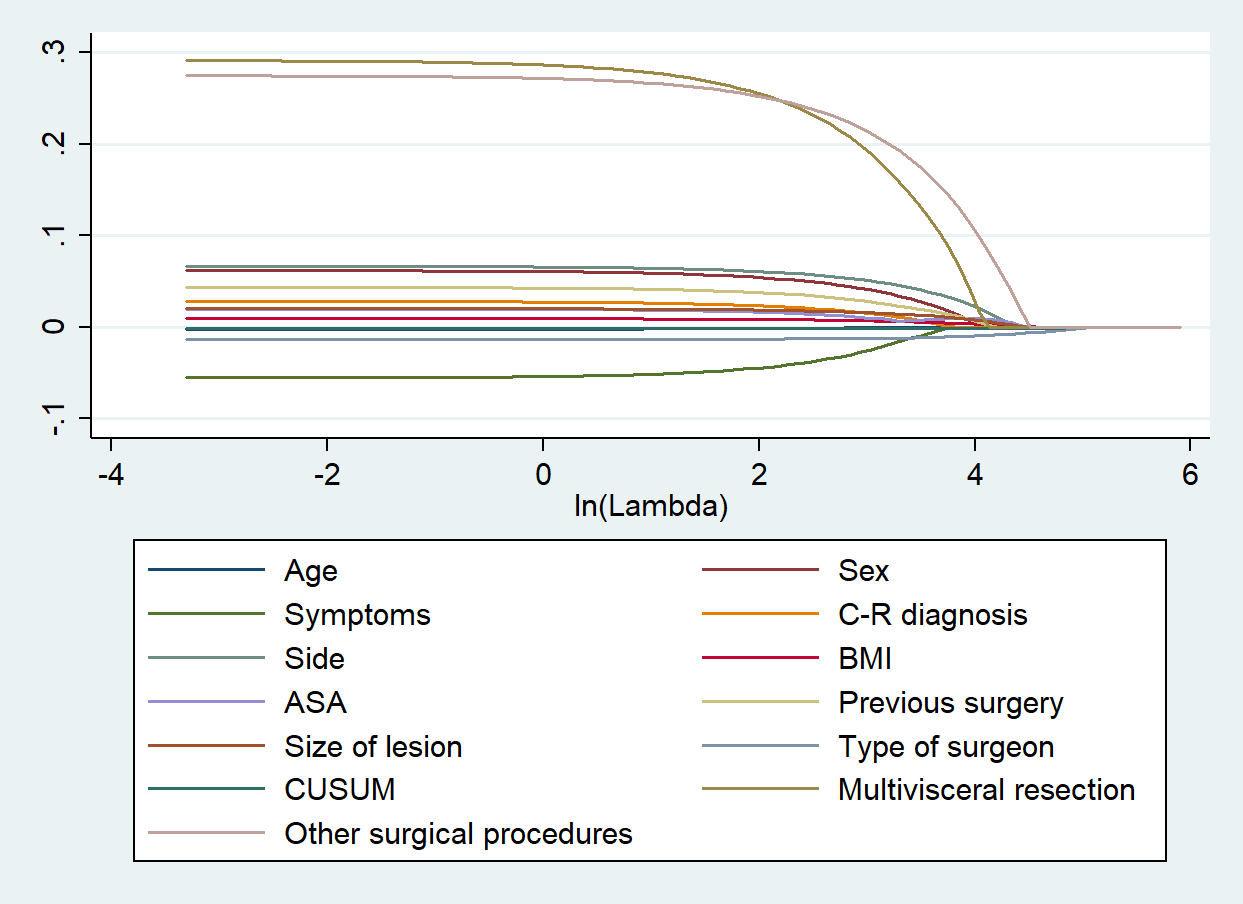


**Legend:** Each line represents a potential regressor; the x-axis represents the LASSO value; the y-axis represents the Lambda value in logarithmic form. The regressor was included if the LASSO value was >0 for the optimal Lambda value. The optimal Lambda values were 0.037 for model A; ASA= American Society of Anesthesiologists score; C-R= Clinical and radiological diagnosis; BMI= Body Mass index; CUSUM= Cumulative sum of procedures.

**Supplementary Figures 1 -** Least absolute shrinkage and selection operator (LASSO) method to preselect the variables - **Panel B-** Model B, conversion


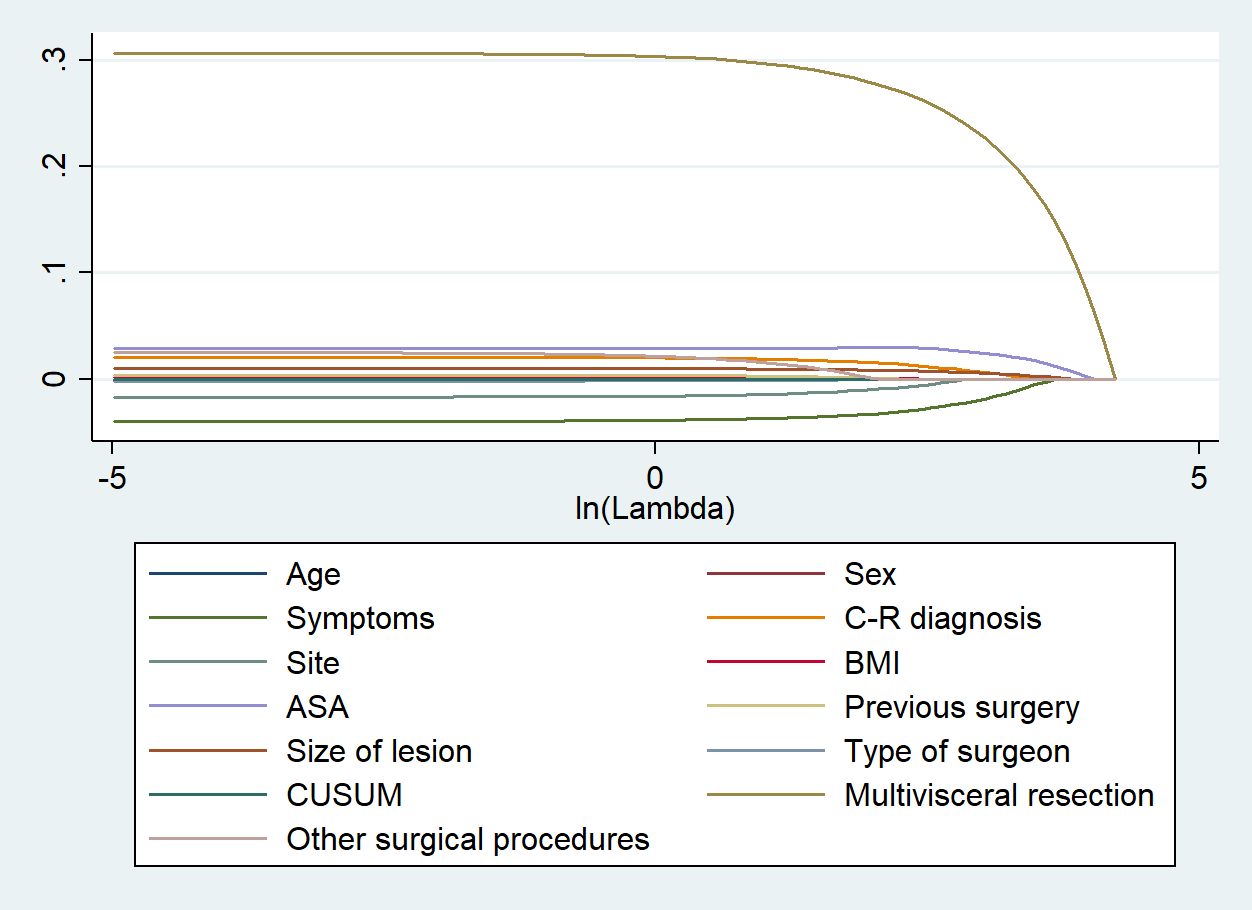


**Legend:** Each line represents a potential regressor; the x-axis represents the LASSO value; the y-axis represents the Lambda value in logarithmic form. The regressor was included if the LASSO value was >0 for the optimal Lambda value. The optimal Lambda values were 6.113 for model B; ASA= American Society of Anesthesiologists score; C-R= Clinical and radiological diagnosis; BMI= Body Mass index; CUSUM= Cumulative sum of procedures.

**Supplementary Figures 1 -** Least absolute shrinkage and selection operator (LASSO) method to preselect the variables - **Panel C-** Model C, operative time over 75^th^ percentile or conversion


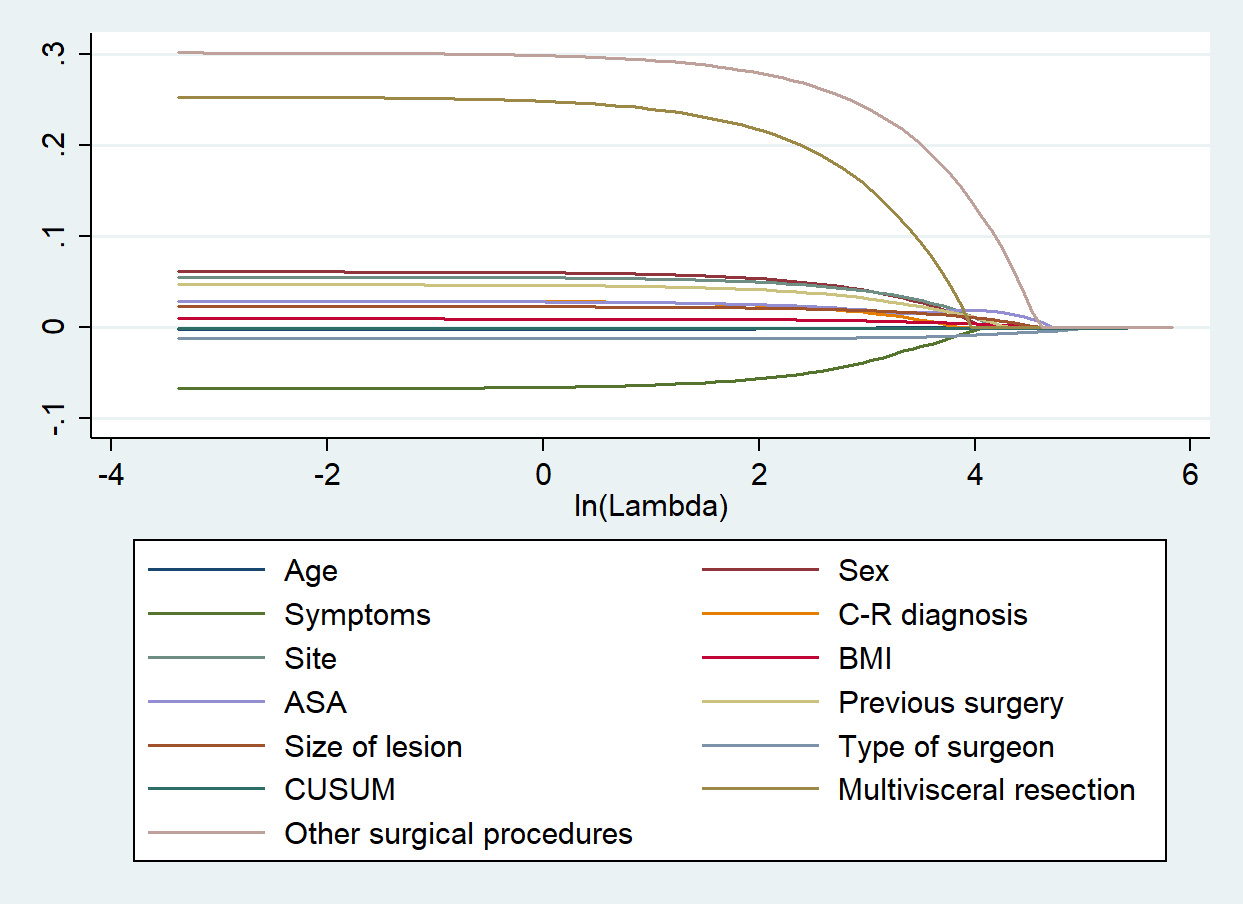


**Legend:** Each line represents a potential regressor; the x-axis represents the LASSO value; the y-axis represents the Lambda value in logarithmic form. The regressor was included if the LASSO value was >0 for the optimal Lambda value. The optimal Lambda values were 0.034 for model C; ASA= American Society of Anesthesiologists score; C-R= Clinical and radiological diagnosis; BMI= Body Mass index; CUSUM= Cumulative sum of procedures.

**Supplementary Figures 2-** Receiver operating characteristic (ROC) curves for model A in the training cohort

**
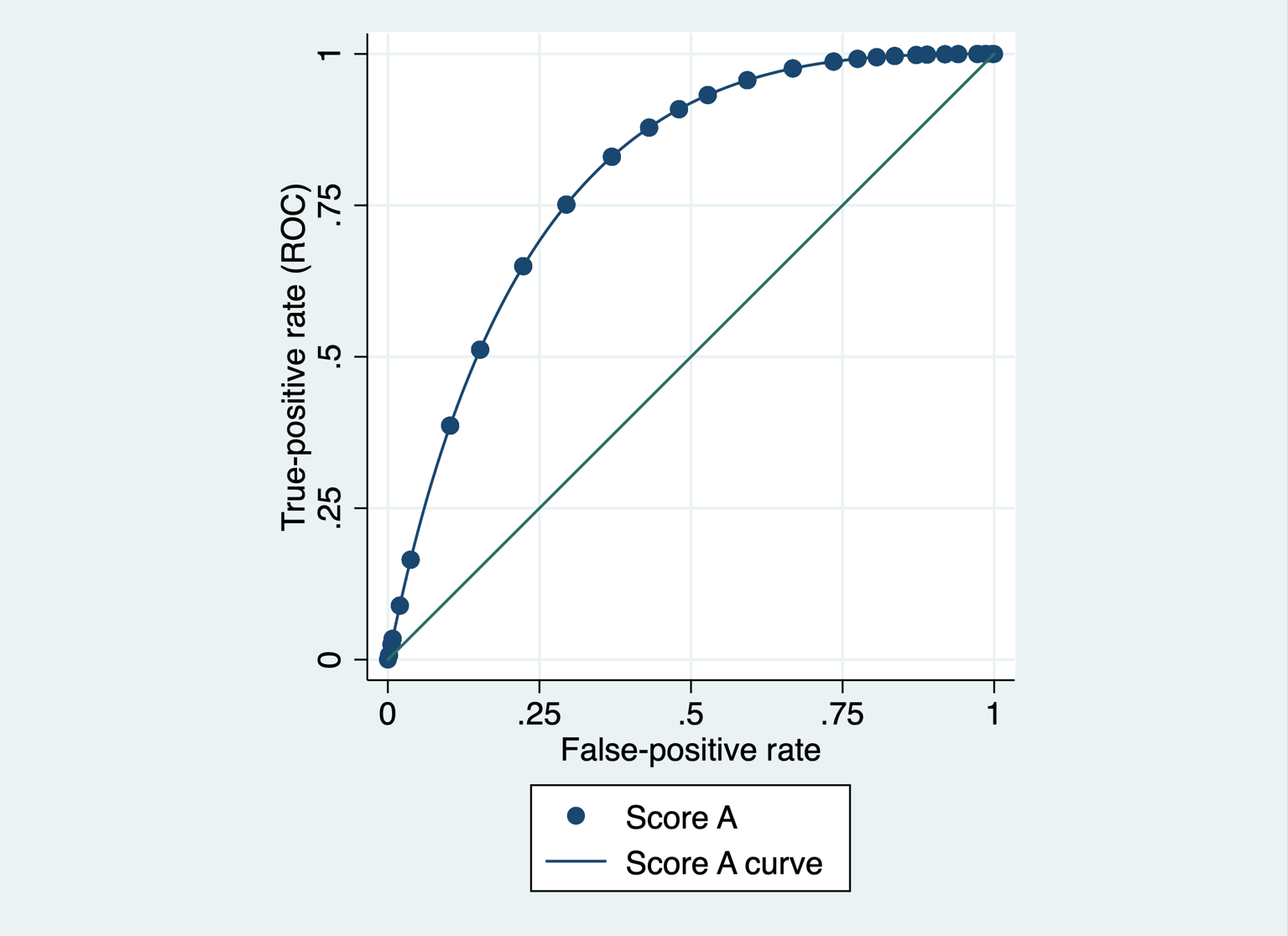
**

**Legend**: **Panel A-** Model A, operative time over 75^th^ percentile

**Supplementary Figures 2-** Receiver operating characteristic (ROC) curves for model B in the training cohort

**
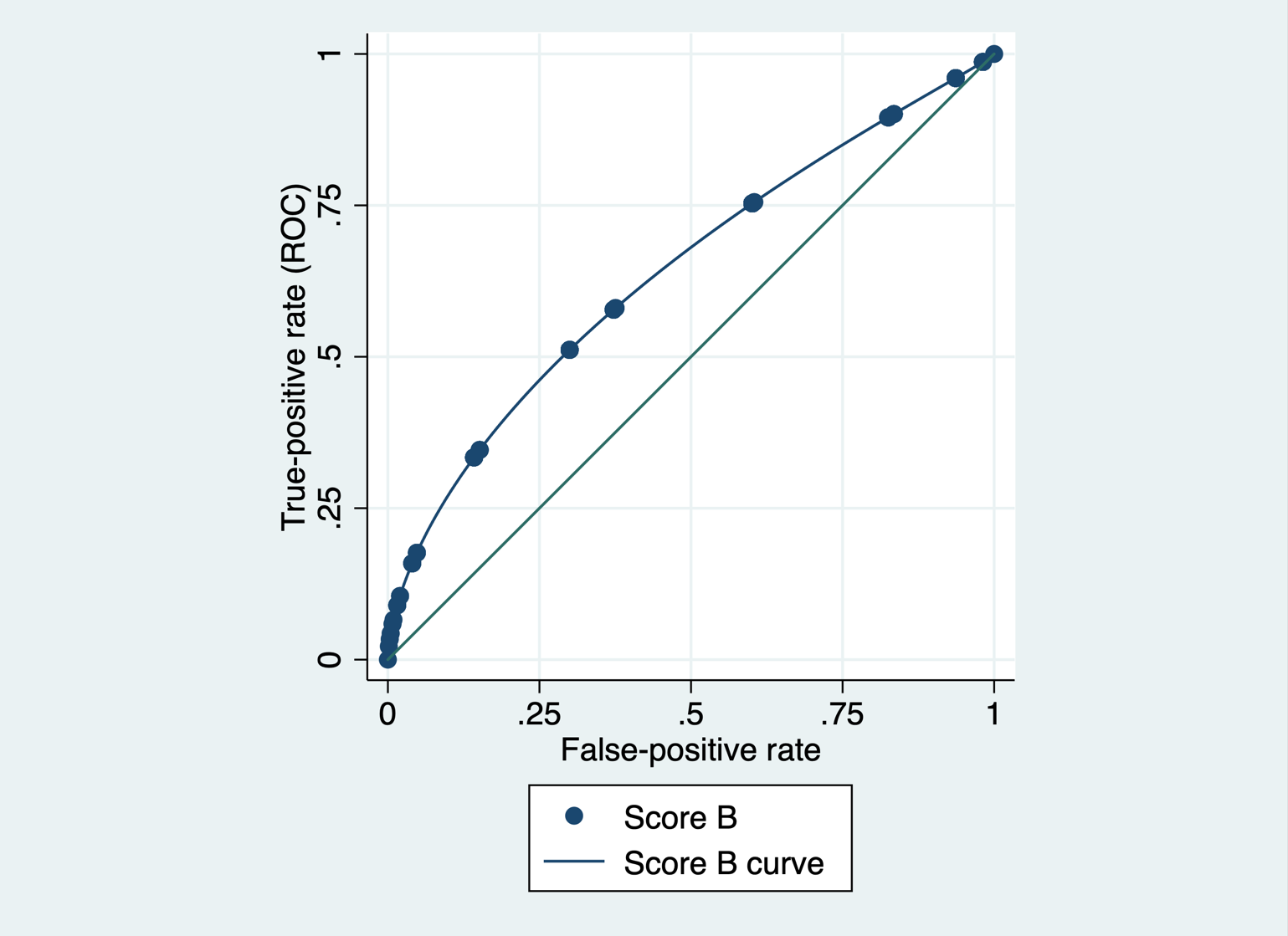
**

**Panel B-** Model B, conversion

**Supplementary Figures 2-** Receiver operating characteristic (ROC) curves for model C in the training cohort

**
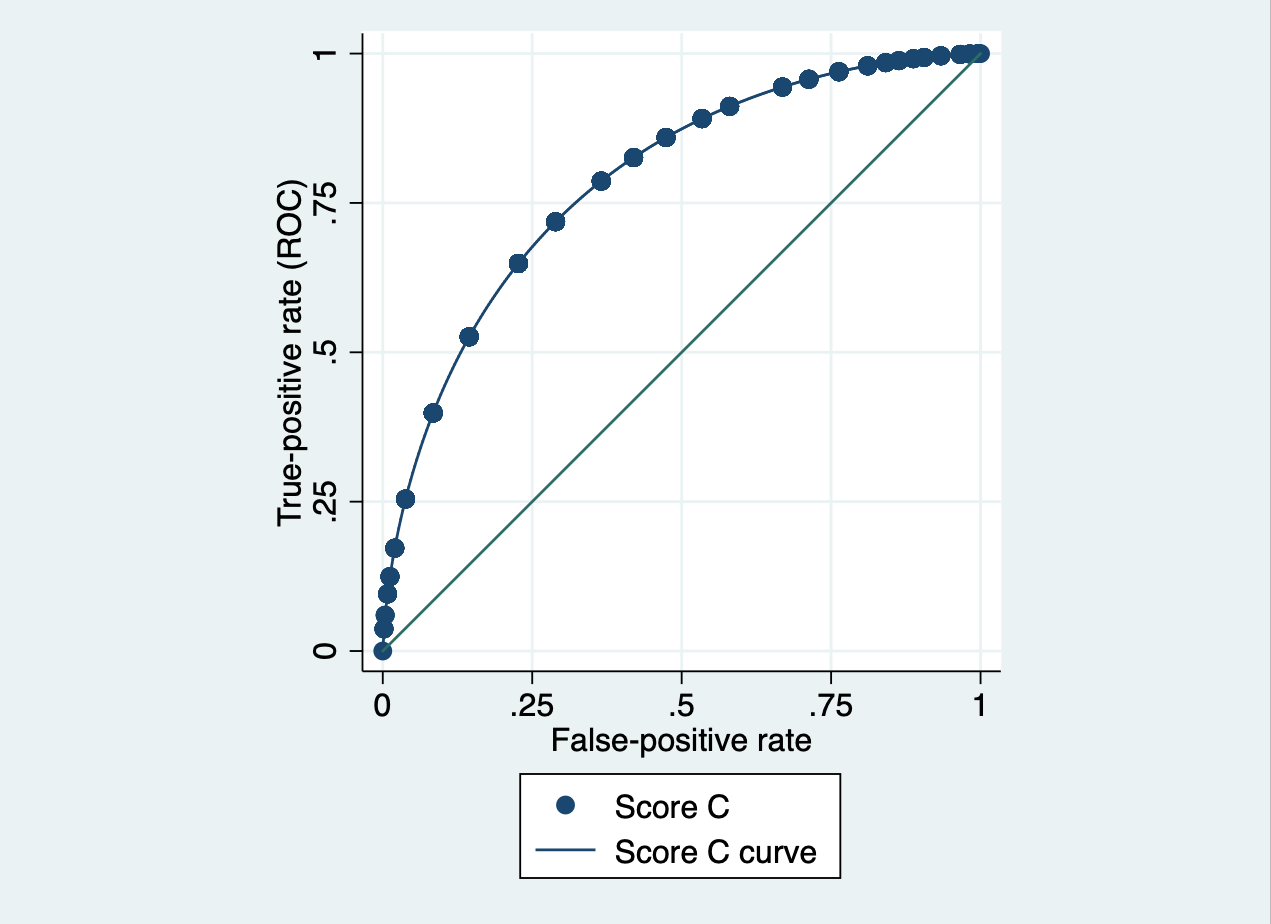
**

**Panel C-** Model C, operative time over 75^th^ percentile or conversion

**Supplementary Figures 3 –** Calibration curve in validation cohort; **Panel A-** Model A, operative time over 75^th^ percentile.

**
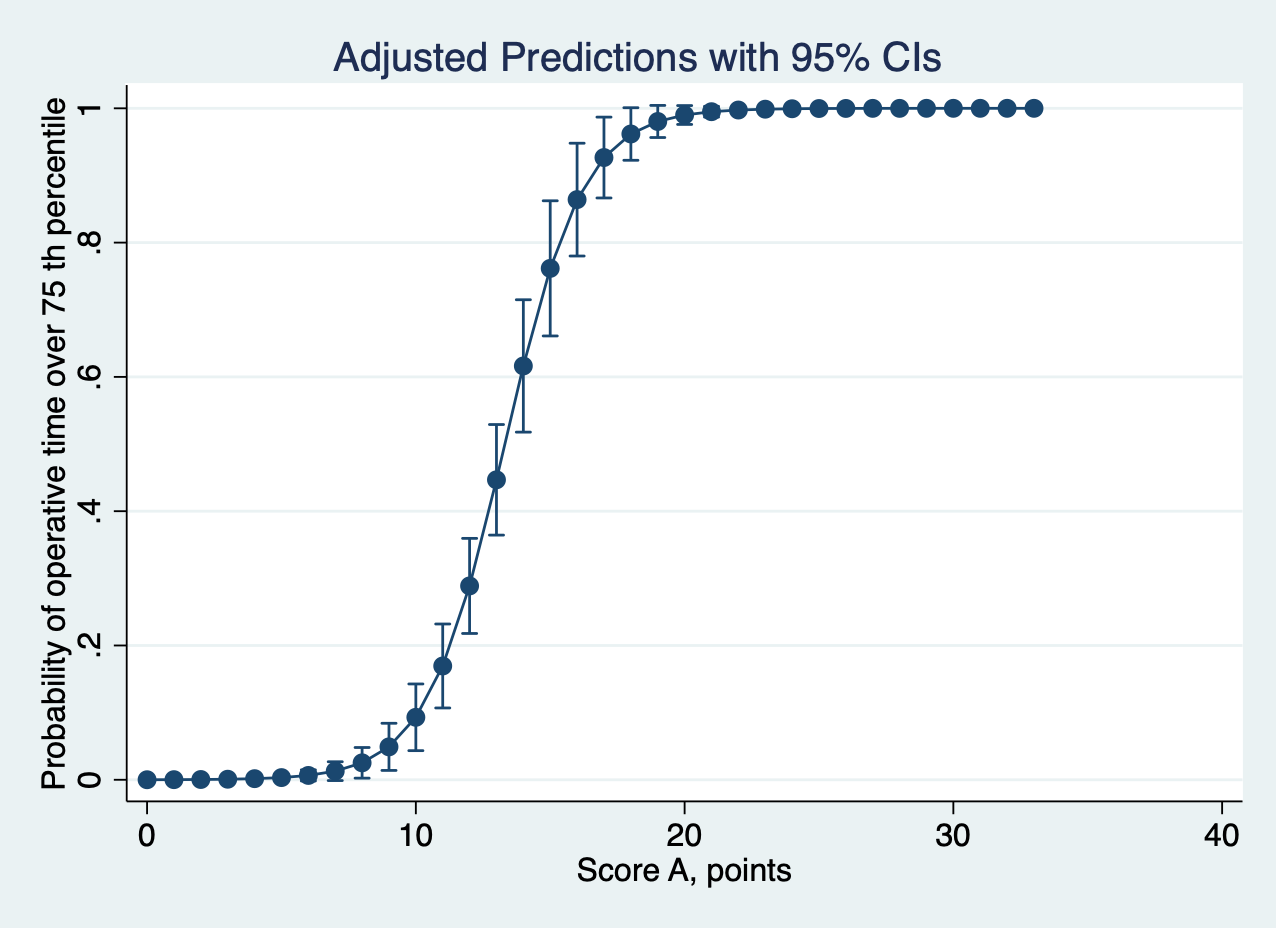
**

**Legend:** The blue line the trend of the score; in the x-axis, all score values are reported; the y-axis represents the target event's probability. Each score value (blue circle) was graphically reported with 95% of interval confidence (whiskers). The calibration demonstrates that the probability of an operative time above the 75^th^ percentile (140 minutes) becomes significant only for score values ​​greater than 9 and reaches 99% for Score A values ​​equal to 21. For values greater than 21, the increased probability of an operative time above the 75^th^ percentile is statistically significant but clinically non-relevant.

**Supplementary Figures 3 –** Calibration curve in validation cohort; **Panel B-** Model B, conversion.

**
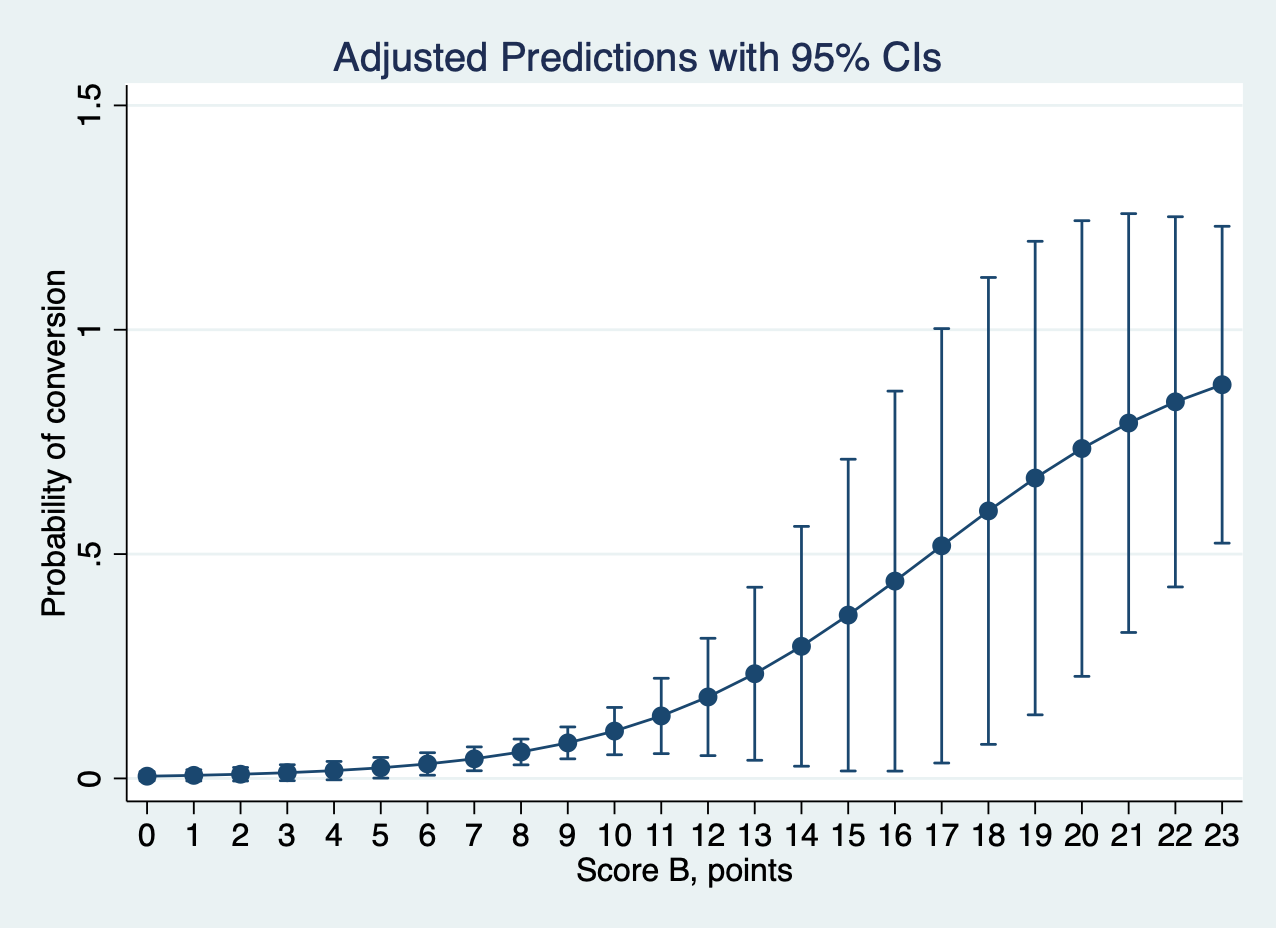
**

**Legend:** The blue line the trend of the score; in the x-axis, all score values are reported; the y-axis represents the target event's probability. Each score value (blue circle) was graphically reported with 95% of interval confidence (whiskers). The calibration demonstrates that the probability of cLA becomes significant only for score values greater than 6 and reaches 84% for Score B values ​​equal to 23

**Supplementary Figures 3 –** Calibration curve in validation cohort; **Panel C-** Model C, operative time over 75^th^ percentile or conversion.

**
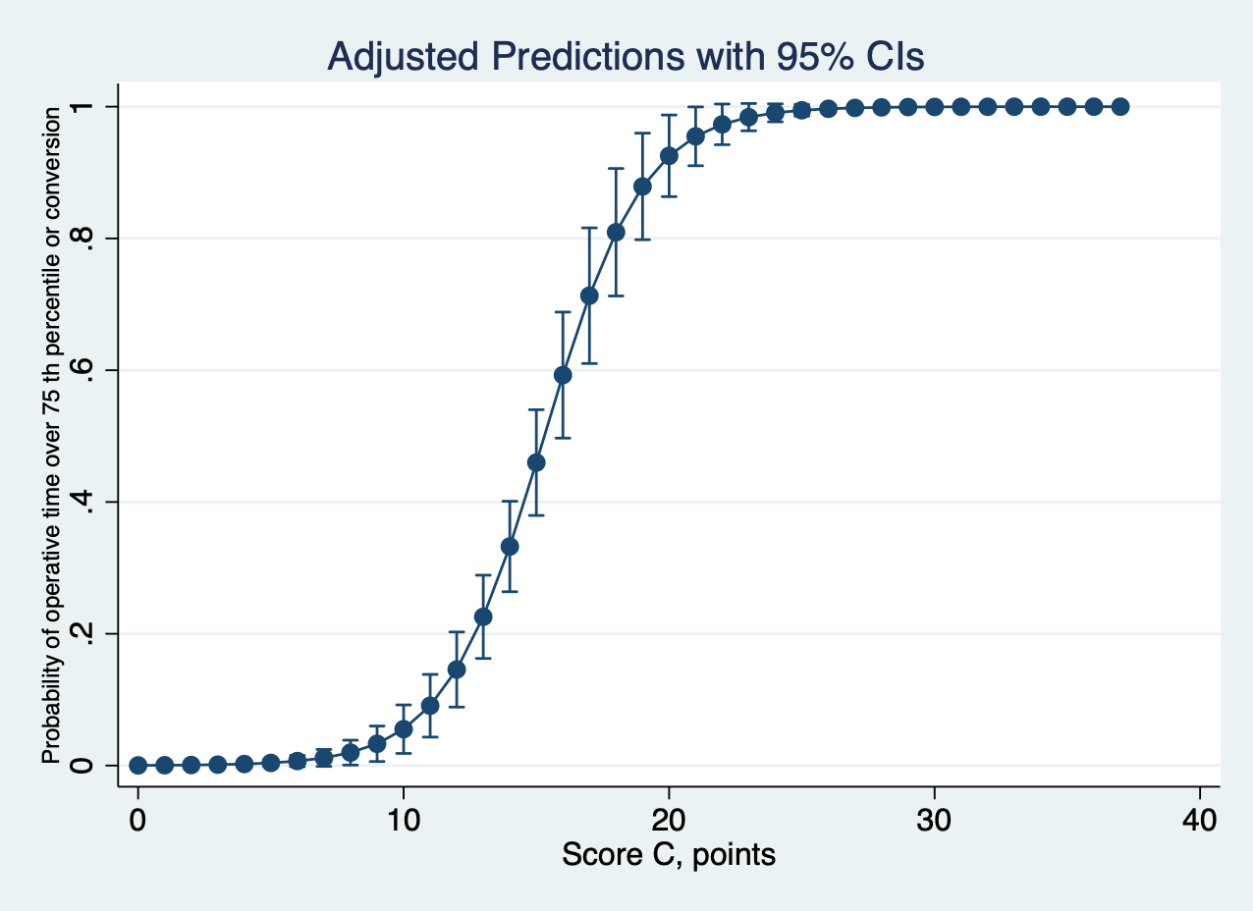
**

**Legend:** The blue line the trend of the score; in the x-axis, all score values are reported; the y-axis represents the target event's probability. Each score value (blue circle) was graphically reported with 95% of interval confidence (whiskers). The calibration demonstrates that the probability of a pOT or cLA becomes significant only for score values greater than 8 and reaches 99% for Score C values ​​equal to 25. For values greater than 25, the increased probability of a pOT or cLA is statistically significant but clinically non-relevant

**Supplementary Figures 4-** Receiver operating characteristic (ROC) curves for model A (Clavien-Dindo class >2) in the validation cohort

**
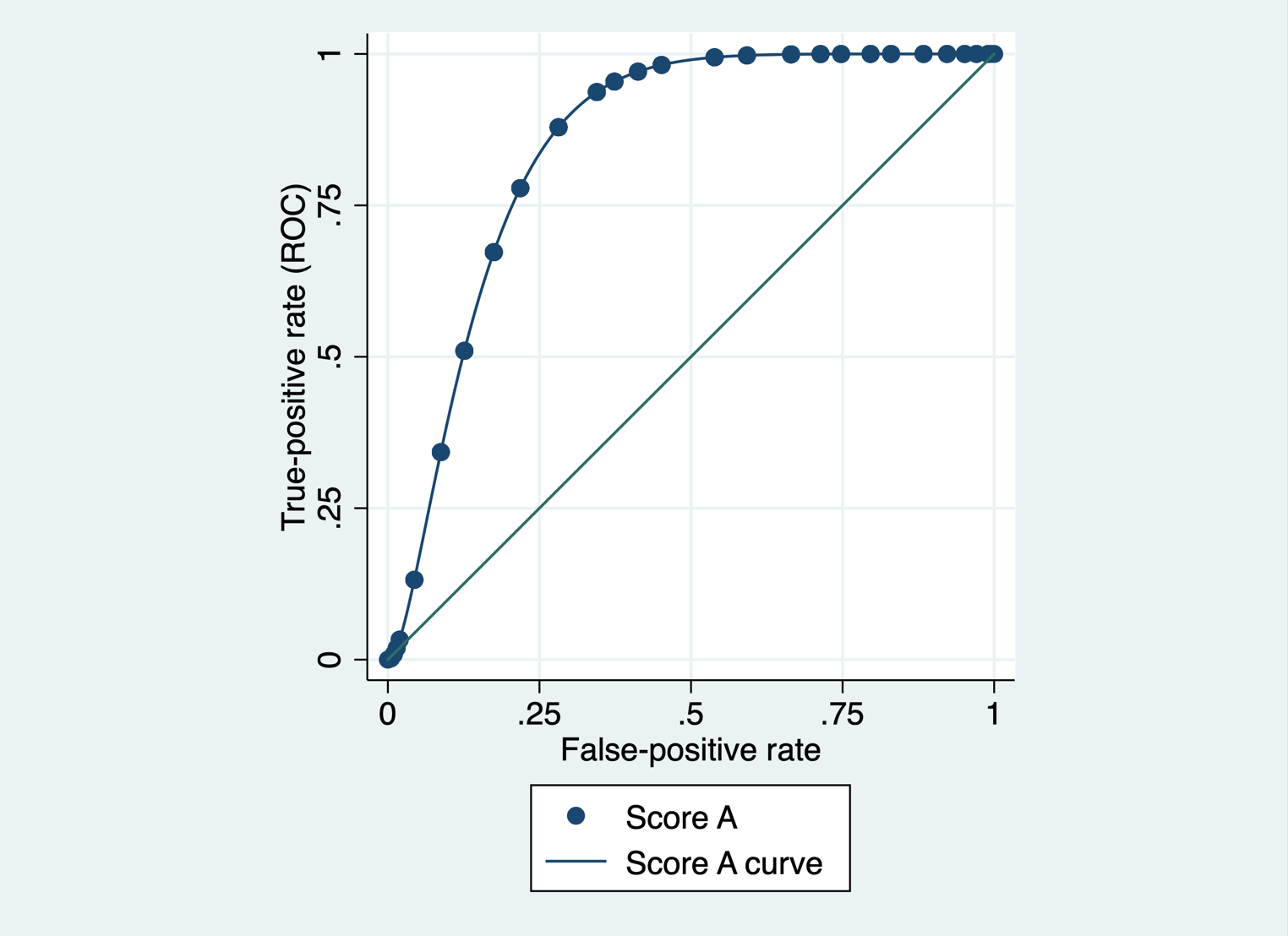
**

**Legend**: **Panel A-** Model A, operative time over 75^th^ percentile;

**Supplementary Figures 4-** Receiver operating characteristic (ROC) curves for model B (Clavien-Dindo class >2) in the validation cohort

**
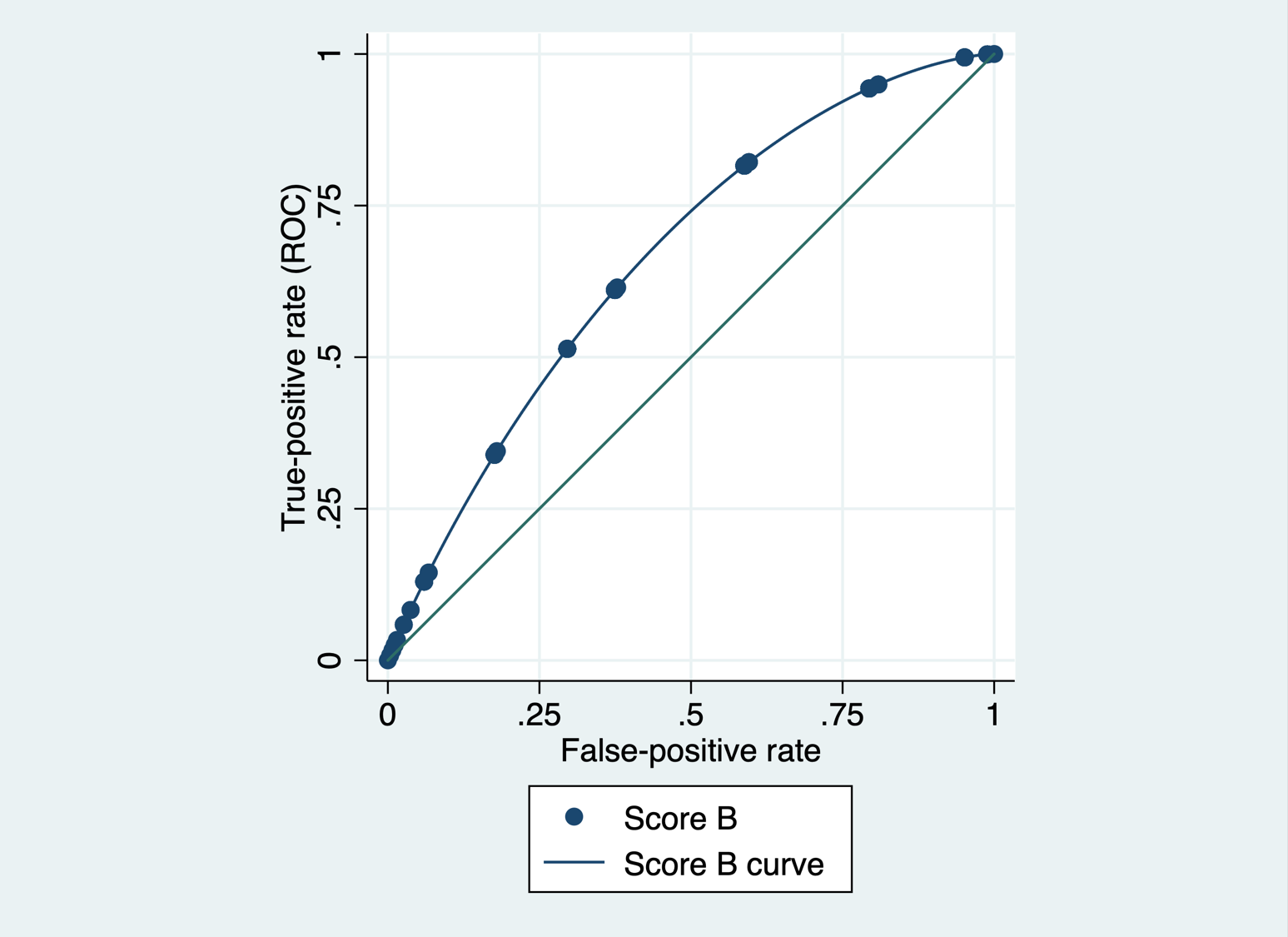
**

**Panel B-** Model B, conversion

**Supplementary Figures 4-** Receiver operating characteristic (ROC) curves for model C (Clavien-Dindo class >2) in the validation cohort


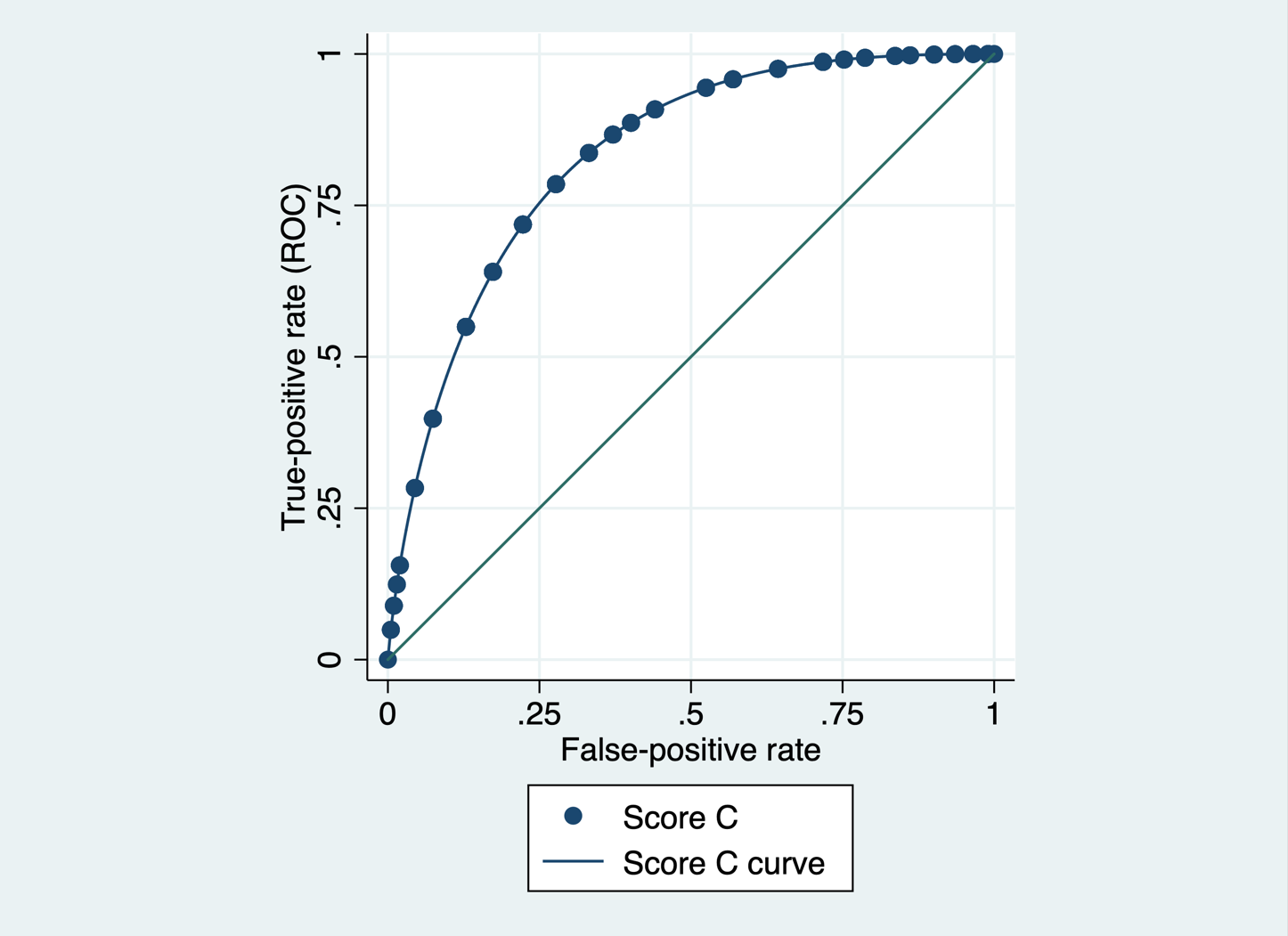


**Panel C-** Model C, operative time over 75^th^ percentile or conversion
